# Intra-Arrest Administration of Cyclosporine and Methylprednisolone Does Not Reduce Postarrest Myocardial Dysfunction

**DOI:** 10.1155/2019/6539050

**Published:** 2019-06-11

**Authors:** Meshe Chonde, Katharyn L. Flickinger, Matthew L. Sundermann, Allison C. Koller, David D. Salcido, Cameron Dezfulian, James J. Menegazzi, Jonathan Elmer

**Affiliations:** ^1^UPMC Heart and Vascular Institute, Department of Cardiology, USA; ^2^University of Pittsburgh, Department of Emergency Medicine, USA; ^3^University of Pittsburgh, Department of Critical Care Medicine, USA

## Abstract

**Objective:**

To determine whether the administration of intra-arrest cyclosporine (CCY) and methylprednisolone (MP) preserves left ventricular ejection fraction (LVEF) and cardiac output (CO) after return of spontaneous circulation (ROSC).

**Methods:**

Eleven, 25-30kg female swine were randomized to receive 10mg/kg CCY + 40mg MP or placebo, anesthetized and given a transthoracic shock to induce ventricular fibrillation. After 8 minutes, standard CPR was started. After two additional minutes, the experimental agent was administered. Animals with ROSC were supported for up to 12h with norepinephrine as needed. Echocardiography was performed at baseline, and 1, 2, 6 and 12h post-ROSC. Analysis was performed using generalized estimating equations (GEE) after downsampling continuously sampled data to 5 minute epochs.

**Results:**

Eight animals (64%) achieved ROSC after a median of 7 [IQR 5-13] min of CPR, 2 [ IQR 1-3] doses of epinephrine and 2 [IQR 1-5] defibrillation shocks. Animals receiving CCY+MP had higher post ROSC MAP (GEE coefficient -10.2, P = <0.01), but reduced cardiac output (GEE coefficient 0.8, P = <0.01) compared to placebo. There was no difference in LVEF or vasopressor use between arms.

**Conclusions:**

Intra-arrest cyclosporine and methylprednisolone decreased post-arrest cardiac output and increased mean arterial pressure without affecting left ventricular ejection fraction.

## 1. Introduction

Each year, more than 500,000 cardiac arrests occur in the United States alone, and patient outcomes remain unacceptably poor [1]. Many patients that achieve return of spontaneous circulation (ROSC) develop subsequent hemodynamic instability, macro- and microcirculatory dysfunction, and end organ damage, a process that has been broadly described as the “postresuscitation syndrome” [2, 3]. Significant left ventricular systolic and diastolic dysfunction during this period is common and has been termed postarrest myocardial dysfunction (PAMD) [4]. Observational studies have suggested an association between PAMD and worse patient outcomes [5, 6].

The pathogenesis of PAMD involves ischemia-reperfusion injury, activation of the inflammatory cascade, and elevated levels of circulating catecholamines, all of which can worsen myocardial function [2, 7, 8]. The complexity of these interdependent pathways makes it unlikely that a single drug will be an effective therapy [9]. Rather, a cocktail of several agents with different targets may be necessary. One promising potential drug combination that targets multiple pathways thought to contribute to PAMD is cyclosporine (CCY) and methylprednisolone (MP). CCY blocks the mitochondrial permeability transition pore thereby preventing apoptosis and cell death [10] and improving contractility [11, 12], while MP decreases macrophage and leukocyte activation [13]. Both agents also decrease inflammatory cytokine production [10, 14–19].

Several preclinical studies have suggested that CCY [10, 15–18] or corticosteroids [20–22] attenuate PAMD when tested individually. However, these studies have measured cardiovascular function only briefly after return of spontaneous circulation (ROSC). Moreover, the effect of CCY and MP in combination has never been evaluated. Thus, we sought to examine the effect of combination treatment on longer-term myocardial function after ROSC. Our primary hypothesis was that CCY+MP would reduce PAMD compared to placebo. Our secondary hypothesis was that CCY+MP would decrease the inflammatory response compared to placebo.

## 2. Methods

All aspects of this study were reviewed and approved by the University of Pittsburgh Institutional Animal Care and Use Committee. The experiment was conducted in compliance with the NIH Guide for the Care and Use of Animals.

### 2.1. Animal Preparation

We sedated 11 female, mixed breed swine, weighing 25-35kg with intramuscular ketamine (10 mg/kg) and xylazine (4 mg/kg). Once sedated, we placed a 20 g intravenous catheter in a peripheral ear vein, administered a 50mcg/kg bolus of IV fentanyl and 5 mg vecuronium, and intubated the trachea with direct laryngoscopy and a 5-0 cuffed endotracheal tube. We then initiated and titrated continuous infusions of intravenous fentanyl (starting dose 200mcg/kg/hr) and midazolam (starting dose 4 mg/hr) to maintain a surgical plane of anesthesia. We administered 1 g ceftriaxone and 500 mg metronidazole to minimize risk of sepsis from instrumentation and periarrest translocation of gastrointestinal flora into the blood stream.

We ventilated the animals with an Ohmeda 7000 ventilator (Ohmeda, BOC Health Care, Madison, WI), initial tidal volumes of 10 mL/kg, respiratory rates of 12-16 breaths per minute and 21% oxygen. We titrated respiratory rate to maintain an end-tidal carbon dioxide between 35 and 45mmHg (Zoll M series CCT capnometer) and inspired oxygen to maintain arterial oxygen saturations >94%. We used standard Lead II electrocardiogram (ECG) configuration for cardiac monitoring. We placed 9 French femoral arterial and venous introducer sheaths via direct cut-down. Through the arterial sheath, we introduced a micromanometer-tipped catheter (Millar, Inc. Houston TX) into the descending aorta. Through the venous sheath, we introduced a Continuous Cardiac Output (CCO) thermodilution catheter (Edwards LifeScience, Irvine CA) into the pulmonary artery. We confirmed proper positioning of the catheters by inspection of the hemodynamic pressure tracings. We used a Vigilance II monitor (Edwards LifeScience) to calculate cardiac output from the CCO catheter. We measured and recorded all physiological parameters continuously using LabChart (AD Instruments, Colorado Springs CO).

We randomized animals in permuted blocks of 2 and 4 to study agent or placebo. A third party not involved in the experiment made the allocation tables and placed treatment assignments in opaque envelopes. Prior to inducing ventricular fibrillation (VF), an unblinded laboratory technician in a different room opened the envelope corresponding to the experiment number and prepared two opaque syringes containing 10 mg/kg of cyclosporine A (Sandimmune, Novartis) in a 10 mL syringe and 40 mg methylprednisolone (Solumedrol, Pfizer, NY) reconstituted in 5 mL normal saline, or two syringes with equal volumes of saline (placebo).

We recorded instrumentation time as the duration from the initial bolus of fentanyl until VF was induced. We attempt to standardize this interval by arresting the animal as close to 60 minutes as possible. We induced VF by delivering a 60 Hz, 100 mA, alternating current shock via external transthoracic electrodes for three seconds. We allowed 8 minutes of untreated VF, after which we began closed-chest CPR with a mechanical compression device (LUCAS™, JOLIFE AB, Sweden) at a rate of 100 compressions per minute.

After 2 minutes of compressions (10 minutes from VF induction), we administered study drugs or placebo, as well as 40U vasopressin, 1 mEq/kg sodium bicarbonate, and 0.1mg/kg epinephrine. We continued compressions for another 3 minutes and then performed pulse and rhythm checks at 2-minute intervals. If the animal remained pulseless then epinephrine 0.015mg/kg bolus was administered and repeated every 4 minutes as needed and CPR continued. If VF or ventricular tachycardia were noted, we attempted defibrillation with a single 150J biphasic shock. We continued CPR until ROSC or until 20 minutes without successful resuscitation.

### 2.2. Postresuscitation Management

Animals that achieved ROSC received standardized postresuscitation critical care for 12 hours, with echocardiography and blood sampling at predetermined time intervals. Briefly, we titrated fentanyl and midazolam to maintain a surgical plane of anesthesia. If mean arterial pressure (MAP) fell below 65 mmHg, we checked for predicted fluid responsiveness by assessing pulse pressure variation with the respiratory cycle. We bolused hypotensive, fluid responsive animals with 10 ml/kg Lactated Ringer's, which we repeated until the animal was normotensive or no longer fluid responsive. We treated hypotensive animals that were not fluid responsive with a continuous infusion of norepinephrine started at 0.1mcg/kg/min, which we titrated to maintain MAP >65mmHg. We titrated the respiratory rate, oxygen concentration, and positive end expiratory pressure to maintain an arterial oxygen saturation of 94-98% and partial pressure of arterial carbon dioxide 35-40mmHg. We euthanized animals that survived for the 12 hours after ROSC with a rapid IV bolus of 40 mEq potassium chloride.

### 2.3. Data Collection

#### 2.3.1. Transthoracic Echocardiography

We performed two-dimensional echocardiography using a portable transthoracic echocardiogram (Vivid e GE) at baseline (prearrest) and at 1, 2, 3, 6, and 12 hours after ROSC. We obtained left ventricular parasternal long axis views as has been described previously [23]. Two board certified echocardiographers blinded to treatment allocation independently quantified left ventricular ejection fraction and we analyzed the mean of the two reviewers' interpretations.

#### 2.3.2. Blood Gas and Biomarker Collection

We obtained samples of arterial blood at baseline, and then at 1, 2, 3, 6, and 12 hours after ROSC. We analyzed baseline, 1-hour and 6-hour samples, an arterial blood gas analyzer (I-Stat, Heska Copr. Wakesha, WA). We also obtained plasma from samples at all time points by placing the blood in sterile ethylenediaminetetraacetic acid- (EDTA-) treated tubes (BD) that we centrifuged at 5000 rpm for 5 minutes. We allocated plasma into 500uL vials that we kept at −80°C until analysis. We used a Luminex multiplex analyzer to simultaneously measure concentrations of IL-1B, IL-4, IL-8, IL-10, and TNF-alpha using porcine specific antibodies (EMD, Billerica, MA).

#### 2.3.3. Hemodynamics

We measured MAP and CO as described above and recorded these continuous data at 100 Hz using LabChart (AD Instruments, Castle Hill, Australia). We downsampled MAP and CO to mean values in consecutive 5-minute epochs for repeated measures analysis.

#### 2.3.4. Statistical Analysis

We summarized baseline data using descriptive statistics and reported medians with interquartile ranges. We compared repeated measures data using generalized estimating equations (GEEs) with an unstructured covariance matrix and robust standard errors to account for the small sample size of our pilot data. We performed all statistical analyses using Stata v14.2 (StataCORP, College Station, TX) and considered a p-value <0.05 to be statistically significant..

## 3. Results

**Figure 1:**
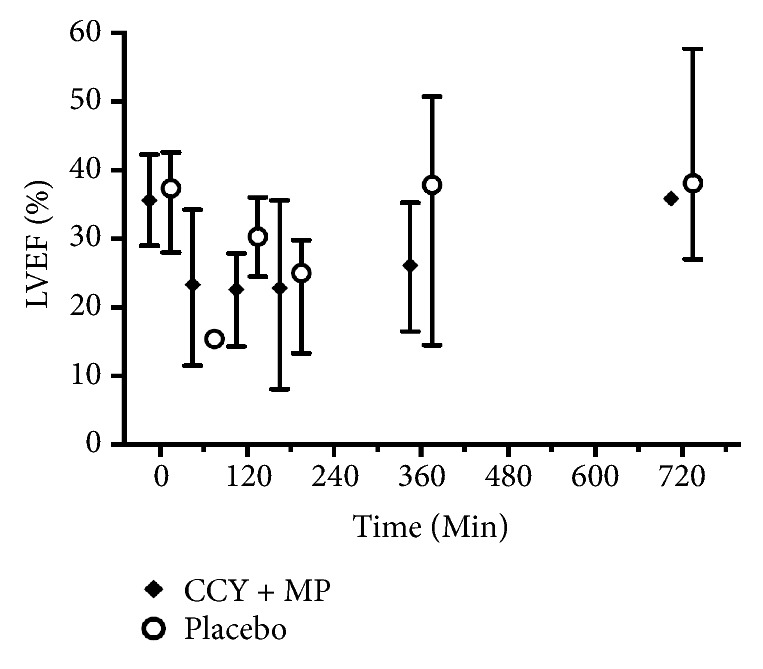
Left ventricular ejection fraction after ROSC. Median values with interquartile range error bars. CCY+MP, cyclosporine, and methylprednisolone; LVEF, left ventricular ejection fraction.

**Figure 2:**
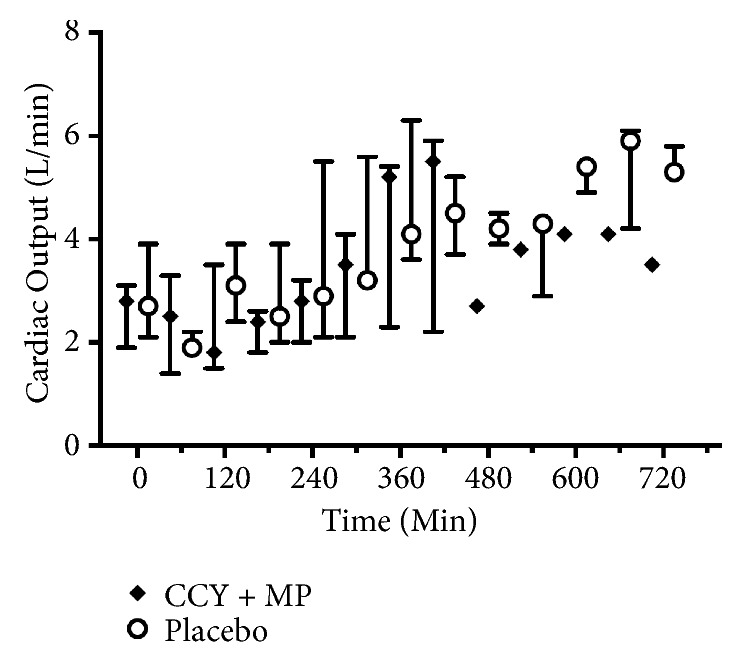
Cardiac output after ROSC. Median values with interquartile range error bars. CCY+MP, cyclosporine methylprednisolone.

**Figure 3:**
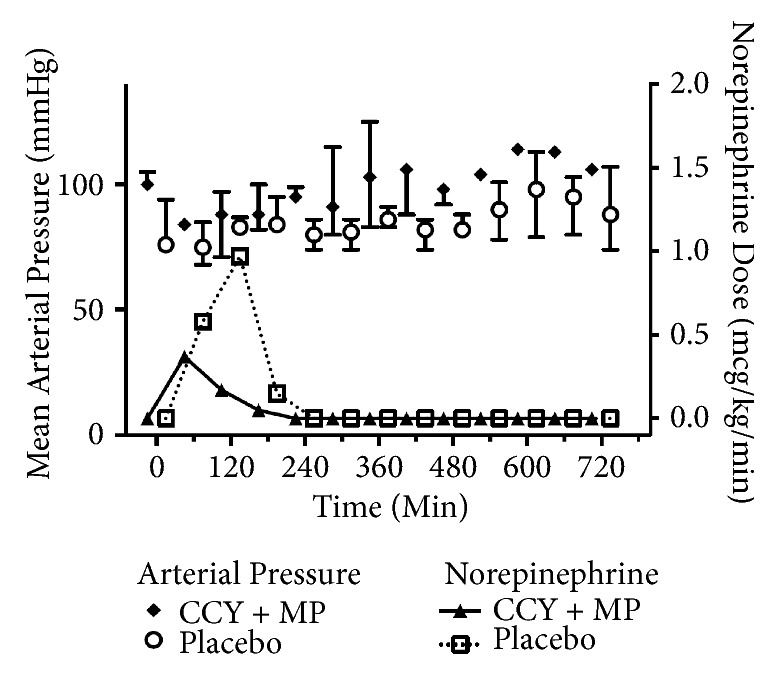
Mean arterial pressure and norepinephrine use after ROSC. Median values with interquartile range error bars. CCY+MP, cyclosporine methylprednisolone.

Baseline parameters were well matched between groups (Table 1). Overall, eleven animals were randomized (5 to CCY+MP and 6 to placebo). One animal randomized to cyclosporine had significant blood loss during femoral vascular access and did not undergo induction of VF or the experimental procedure. This animal was excluded from analysis. 8 animals achieved ROSC (3/4 cyclosporine and 5/6 placebo). In the animals that achieved ROSC, LVEF decreased after arrest with a nadir at 3 h and subsequent return to baseline (Figure 1) but did not differ across treatment groups (GEE coefficient 6.4, p = 0.34). Postarrest CO also initially decreased from baseline (Figure 2) but was significantly higher in the placebo group compared to CCY+MP (GEE coefficient 0.8, P = <0.01). In contrast, MAP was significantly higher in the CCY+MP group compared to placebo (GEE coefficient for MAP -10.2, P = <0.01) with no difference in overall vasopressor requirements, (coefficient for vasopressor dose 0.2, P = 0.26) (Figure 3). However, in the first 4 hours after ROSC, vasopressor requirements were substantially higher in the placebo arm (P <0.01). Levels of IL-1B, IL-8, IL-10, and TNF-alpha did not differ across treatment groups (see supplemental data). However, twenty-seven percent of the plasma assays were contaminated by hemolysis/lipemia, limiting our ability to interpret these cytokine results.

## 4. Discussion

Postarrest myocardial dysfunction (PAMD) is common and worsens survival in those hospitalized after cardiac arrest. Despite its prevalence, effective strategies to attenuate the severity or duration of PAMD have remained elusive. We completed pilot work in a porcine model testing a novel drug combination targeting multiple cellular pathways believed to contribute to PAMD [7]. Our model itself was effective in that we observed a significant decrease in LV ejection fraction and high vasopressor requirements after ROSC in placebo animals. However, PAMD and shock were short-lived, with cardiovascular function returning to baseline by 6 hours after ROSC, and we observed no difference between drug and placebo arms in terms of myocardial function.

While our pilot work was not designed to let us test the independent effects of MP and CCY, we hypothesize that the improvement in vascular tone we observed in the drug arm was due to the MP. Similar effects have been reported in clinical trials testing the utility of corticosteroids after cardiac arrest [24, 25]. Although CCY can cause hypertension [26], this is an effect noted in chronic use rather than an acute-phase effect. It may be that this improved vascular tone in the drug arm reduced cardiac output by increasing afterload. Alternatively, since LV systolic function and cardiac output are increased with norepinephrine [27–29], the trend towards increased vasopressor requirements in the placebo may actually have masked a lower LV ejection fraction and cardiac output. Interestingly, after 6 hours after ROSC, the CCY+MP group had a decrease in cardiac output to baseline, while the placebo group continued to increase. As there was no further vasopressor use, noxious stimulus, or change in sedation this may represent the presence a “secondary insult” such as developing sepsis or delayed hemodynamic response to delayed cellular mediators of systemic inflammation.

Our finding that CCY+MP did not reduce PAMD differs from other preclinical studies that demonstrated an improvement or attenuation of postarrest or postcardiac bypass myocardial function in treatment with cyclosporine [11, 12, 15–18] but is consistent with the results of recent Phase III trials of CCY alone [30]. The dose of cyclosporine previously used in preclinical studies ranged from 2.5mg/kg to 25 mg/kg. We chose 10 mg/kg, as Gill and colleagues [16] demonstrated in a newborn piglet model of asphyxial cardiac arrest that the attenuation of myocardial function and hemodynamics was greatest in those treated with cyclosporine 10 mg/kg compared to 25 mg/kg, 2.5mg/kg, or placebo. Additionally, cyclosporine in our study was administered intra-arrest, prior to ROSC, as suggested by Huang et al. [15], who demonstrated that delay in the administration of cyclosporine until postROSC did not yield significant benefit in attenuation of myocardial dysfunction. If not due to a different dose of medication or delay in administration then what could have played a role in the difference?

Perhaps, cyclosporine is not the right immunosuppressive to use. During the conduct of our work, the Phase III CYRUS trial [30], a multicenter randomized control trial of patients with a nonshockable cardiac arrest treated with intra-arrest cyclosporine demonstrated no improvement in survival, cardiac, or neurologic function. This trial has been criticized in that [9] median time from collapse to cyclosporine administration was at least 19 minutes. Additionally, patients received a dose of cyclosporine 2.5mg/kg intra-arrest, which has been shown as a less effective dose in prior preclinical models [16] and is less than initial daily treatment dose for transplant patients [31], which may have also resulted in blunted effect. Our findings suggest that the neutral results in CYRUS may not be due only to the timing of drug administration or the dose as we observed no protective effects despite a 4-fold higher dose.

From a mechanistic perspective, we found no significant differences in inflammatory cytokine levels across treatment arms. However, several of the samples were contaminated secondary to lipemia and were not suitable for analysis, which may have contributed to our neutral result. Due to our small sample size and limited specimens we were unable to analyze further, but it marks an area of interest for future study.

Our work has several important limitations. First, while our study was prospective, blinded, and the animal subjects randomized in 1:1 or 2:2 blocks, it was intended as pilot work to inform a future, larger study design. Therefore, our sample size was limited, potentially resulting in a Type II error. Although analyses of repeated measures can improve statistical power despite small sample sizes, ultimately our outcomes were still clustered within relatively few animals. Second, our overall ischemic insult during arrest and cardiopulmonary resuscitation was relatively short. This was by design to increase the number of animals achieving ROSC but may have resulted in less severe PAMD. Given the significant decrease in myocardial performance we observed in the hours after ROSC, we do believe that our model should have resulted in a sufficient effect size to test the efficacy of our drug combination but cannot be sure. Indeed, although profound, the PAMD observed in our model was relatively short-lived, unlike PAMD observed in the clinical setting which may persist for hours to days [5, 32]. Compared to our model, Kern and colleagues saw peak PAMD 2 hours after ROSC following 10-minute untreated VF and 6 hours after ROSC following 15-minute VF [33]. It may be that the combination of CCY+MP would have greater efficacy in a more severe model of injury with a greater systemic inflammatory and catecholamine response. Finally, to maintain transparency, we report the results of our biomarker assays which may have been limited by smaller sample size due to lipemia and hemolysis but represent an area for future investigation.

## 5. Conclusion

Our pilot work does not support pursuing combined intra-arrest administration of cyclosporine and methylprednisolone as a strategy to reduce postarrest myocardial dysfunction.

## Figures and Tables

**Table 1:** Baseline Characteristics.

	CCY + MP (n = 5)	Control (n = 6)
*Hemodynamics*		
MAP (mmHg)	88 (88-96)	85 (74-92)
CO (L/min)	2.8 (1.9-3.1)	2.7 (2.1-3.9)
*Arrest characteristics*		
Weight (kg)	25.0 (24.4-27.5)	24.2 (23.8-25.4)
CPR duration (min)	7 (5-7)	7 (5-13)
Countershocks (#)	2 (1.5-2)	2 (1-5)
Epinephrine bolus (#)	2	2 (1-3)
*Laboratory values*		
pH	7.56 (7.54-7.58)	7.55 (7.49-7.60)
pCO2 (mmHg)	38 (37-40)	32 (31-38)
pO2 (mmHg)	89 (88-240)	93 (77-107)
HCO3 (meq/L)	33.9 (33.7-37.1)	29.8 (29.6-32.4)
Sp02 (%)	98 (98-100)	99 (96-99)
Na (meq/L)	140 (140-142)	142 (141-142)
K (meq/L)	3.5 (3.1-3.7)	3.4 (3.2-3.7)
Ca (meq/L)	1.35 (1.28-1.39)	1.22 (1.17-1.34)
Glu (mmol/L)	119 (110-125)	102 (96-111)
Hct	25 (25-27)	25 (24-26)
*Echocardiographic parameters*		
LVEDD (cm)	3.5 (3.3-3.6)	2.9 (2.9-3.0)
LVESD (cm)	2.7 (2.4-2.9)	2.1 (2.0-2.2)
Fractional Shortening (%)	23.9 (19.3-28.5)	26.9 (24.3-32.8)
LVEF (%)	35.6 (29.0-42.3)	37.5 (328.0-42.6)

Data is shown as median (IQR). CO, cardiac output; Glu, glucose; Hct, Hematocrit; LVEDD, left ventricular end diastolic diameter; LVEF, left ventricular ejection fraction; LVESD, left ventricular end systolic diameter; MAP, mean arterial pressure; ROSC, return of spontaneous circulation

## Data Availability

The data used to support the findings of this study are included within the article.

## Abbreviations

CCY: Cyclosporine A
CO: Cardiac output
EDTA: Ethylenediaminetetraacetic acid
IL-1*β*: Interleukin-1*β*
IL-10: Interleukin-10
IL-4: Interleukin-4
IL-8: Interleukin-8
LVEDD: Left ventricular end diastolic diameter
LVESD: Left ventricular end systolic diameter
LVEF: Left ventricular ejection fraction
MAP: Mean arterial pressure
MP: Methylprednisolone
ROSC: Return of spontaneous circulation
TNF-*α*: Tumor necrosis factor-*α*.

## Supplementary Materials

Supplementary Materials Biomarker Levels after ROSC. Median values with interquartile range error bars. CCY+MP, cyclosporine, and methylprednisolone; IL, interleukin; TNF, tumor necrosis factor.
