# Supplementary material for: Intra-Arrest Administration of Cyclosporine and Methylprednisolone Does Not Reduce Postarrest Myocardial Dysfunction
Source: Biomed Res Int. 2019 Jun 11;2019:6539050. doi: 10.1155/2019/6539050 (PMC6594305; doi:10.1155/2019/6539050)
Supplement: Supplementary Materials — Biomarker Levels after ROSC. Median values with interquartile range error bars. CCY+MP, cyclosporine, and methylprednisolone; IL, interleukin; TNF, tumor necrosis factor. [file 6539050.f1.pdf]

Appendix 1 Supplemental Data. Biomarker Levels

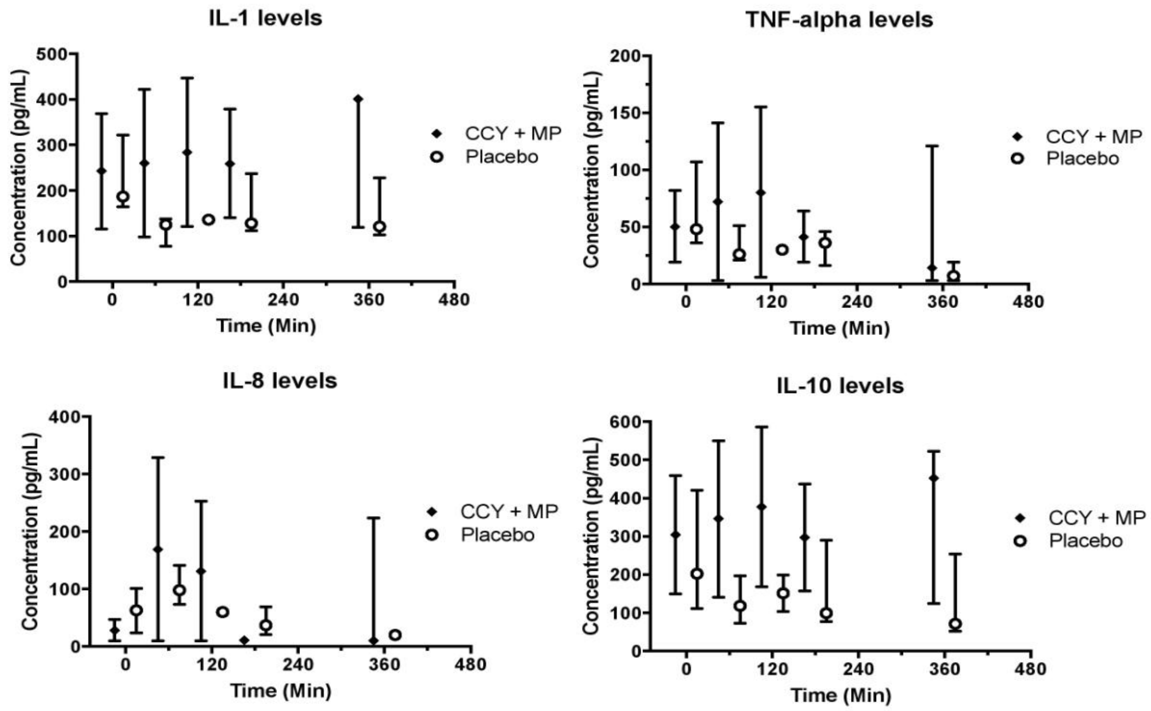

Supplemental Data. Biomarker Levels after ROSC. Median values with interquartile range error bars. CCY+MP, cyclosporine and methylprednisolone; IL, interluekin; TNF, tumor necrosis factor
